# Impact of PET/CT Imaging with FDG in Locally Advanced Cervical Carcinoma—A Literature Review

**DOI:** 10.3390/curroncol31050188

**Published:** 2024-04-29

**Authors:** Ottó Molnar, Oreste Mihai Straciuc, Simona Mihuțiu, Liviu Lazăr

**Affiliations:** 1 Doctoral Studies Department, Biomedical Science, 410087 Oradea, Romania; 2 Centrul PET/CT Pozitron Diagnosztika, 410035 Oradea, Romania; 3 Department of Medicine-Psycho-Neuroscience and Recovery, Faculty of Medicine and Pharmacy, 410073 Oradea, Romania; 4 Oncology Department, Pelican Hospital, 410469 Oradea, Romania; 5 Băile Felix Medical Rehabilitation Hospital, 417500 Băile Felix, Romania

**Keywords:** cervical cancer, PET, computed tomography, FDG, oncology, radiopharmaceuticals, diagnostics, screening, treatment, follow up

## Abstract

Positron emission tomography (PET) and computed tomography (CT) have evolved as a pivotal diagnostic modality in the field of oncology. With its increasing application in staging and ready availability, it becomes imperative for committed radiation oncologists to possess a complete analysis and understanding of integration of molecular imaging, which can be helpful for radiation planning, while also acknowledging its possible limitations and challenges. A significant obstacle lies in the synthesis and design of tumor-specific bmolecules for diagnosing and treating cancer. The utilization of radiation in medical biochemistry and biotechnology, encompassing diagnosis, therapy, and control of biological systems, is encapsulated under the umbrella term “nuclear medicine”. Notably, the application of various radioisotopes in pharmaceutics has garnered significant attention, particularly in the realm of delivery systems for drugs, DNA, and imaging agents. The present article provides a comprehensive review of use of novel techniques PET and CT with major positron-emitting radiopharmaceuticals currently in progress or utilized in clinical practice with their integration into imaging and radiation therapy.

## 1. Introduction

Imaging techniques play a key role in medical diagnosis, serving as critical tools for preserving health and life itself. They are indispensable in a myriad of medical investigations, ranging from cancer detection where early diagnosis is imperative to the exploration of neurological diseases and cardiology. Studying metabolic pathways and drug metabolism enhances our understanding of various disorders, potentially paving the way for effective treatments.

Cervical cancer denotes the onset of malignancy originating from cervix cells, the narrow lower portion of the uterus linking it to the vagina (birth canal). Typically, cervical cancer progresses gradually. Preceding its manifestation, cervical cells undergo dysplastic alterations, characterized by the emergence of anomalous cells within cervical tissue. If left unchecked, the abnormal cells may progress into cancerous cells in the cervix and neighboring regions [1].

Cervical carcinoma, when restricted to the lower pelvis, may be managed effectively through either surgical intervention or chemoradiotherapy. For women with less significant tumors (about 2 cm) and lacking uterine involvement, transvaginal radical trachelectomy offers a fertility-preserving alternative. A diverse array of imaging techniques are available, each offering distinct advantages and capabilities for obtaining varying levels of information. These techniques are complementary, spanning from furnishing structural details to molecular insights. Various imaging modalities used for cervical cancer screening are summarized in Table 1. The choice of a particular technique hinges on the desired level of information. For instance, while a ruler suffices for measuring the breadth of a page, it proves inadequate for gauging the length of a football pitch. Different techniques and instrumentation are thus requisite for addressing disparate medical inquiries [2,3,4,5]. 

The PET imaging modality has advantages in its capacity to monitor biochemical and physiological processes within living systems. PET utilizes radiolabeled biomolecules to explore biological processes, with biomolecules ranging from simple entities like O-15-labeled water molecules for studying blood perfusion to complex radiolabeled cells for investigating autoimmune diseases. These techniques enable the measurement of minute molecular quantities without perturbing the biological system due to their sensitivity. Positron emission tomography (PET) and computed tomography are diagnostic imaging modalities that utilize a positron-emitting radionuclide-labeled tracer and hold promise for advancing diagnosis and treatment in gynecological oncology. The image processing techniques, target delineation methodologies, and PET-guided protocols for biologically guided radiation therapy and PET-adaptive therapy are important factors in this regard. The integration of PET with computed tomography (CT) in PET/CT scans enables precise localization of lesions through the accumulation of ^18^F-fluoro-d-glucose (FDG), a radiotracer. PET exhibits superior reliability in diagnosing lymph node metastases compared to conventional imaging modalities such as computed tomography or MRI, positioning it as a valuable non-invasive diagnostic tool [18,19,20,21,22]. The presence of FDG accumulation within lesions serves as an adverse prognostic indicator in uterine cancer cases. Notably, FDG-PET/CT has limited sensitivity for detecting lymphatic metastases in cervical carcinomas having an axis diameter smaller than 5 mm [23]. Consequently, secondary factors such as tumor size or extension into cervical stroma or parametrium are utilized in treatment decision-making. Enhanced imaging techniques contribute to better tumor delineation, aiding in treatment and improving the accuracy of targeted radiation therapy [6,24,25,26,27,28,29,30,31,32,33,34,35,36,37,38]. 

In this review, we will focus in detail on cervical cancer, its causes, types, prevalence, stages of disease, strategies employed for precise diagnosis, therapeutic approaches, and follow-up of treatment response and disease recurrence.

## 2. Materials and Methods

The data from research publications related CT/PET scans were extracted and analyzed for their importance regarding cervical cancer patients. The approach used was to collect information from a large range of existing literature sources, especially PubMed, with related keywords. For this purpose, 127 research papers were selected in which patients were diagnosed positively with different gynecological malignancies, especially cervical cancer, and underwent PET, PET/CT, or PET/MRI evaluation during diagnostic procedures. Those papers which directly emphasize the cervical cancer staging, diagnostics and therapeutics, or treatment were specially taken into consideration. The effect of various labeled compounds on the patients was also taken in account for the review. Input was taken from an expert team of scientists in the field of oncology, nuclear medicine, radiation biology, and therapeutics.

## 3. Cervical Cancer Prevalence 

Cervical cancer is the 4th most prevalent cancer type found among women worldwide and contributes to approximately 7.5% of female cancer-related fatalities. In 2017, there were 12,280 new cases diagnosed and 4210 deaths in the United States due to this cancer. 

Upon presentation of cervical cancer, staging is crucial for treatment planning. Roughly 40% of cervical cancer patients exhibit locally advanced disease, characterized by tumor extension away from cervix into the vagina and lymph nodes [39]. The standard treatment approach for locally advanced cervical cancer typically involves curative-intent therapy comprising concurrent radiation therapy and cisplatin chemotherapy, which includes external beam radiation to the pelvis. Accurate staging is imperative for optimal patient management, as failure to detect disease outside the radiation field may lead to undertreatment, while overtreating undetected disseminated disease can occur. Historically, non-invasive staging techniques such as CT combined with MRI were used to evaluate the extent of local advancement. Cervical cancers are classified into different types based on the specific type of cell where the cancer originated. Squamous cell carcinoma is the most common type, occurring in about 90% of cases, and it develops from cells in the ectocervix (outer part). Adenocarcinoma originates from the glandular cells located in the endocervix (inner part). Clear cell adenocarcinoma, or mesonephroma, is a rare subtype of cervical adenocarcinoma. The preclinical stage of cervical cancer is increasingly detected due to active screening with the Papanicolaou (Pap) test, leading to a substantial reduction in the incidence of the disease by over 50%. Of the estimated 604,000 new cases of cervical cancer diagnosed each year worldwide, human papillomavirus (HPV) types 16 and 18 are responsible for about 71% of cases. An additional 19% of cervical cancers are attributed to HPV types 31, 33, 45, 52, and 58. Research shows that around 90% of HPV infections clear up within two years of onset, with only about 10% persisting. It is still unclear whether the virus is completely eradicated or remains dormant in basal cells with the potential to reactivate later on. Understanding the epidemiology of HPV and its role in causing cancer has led to two key approaches for prevention and early detection: (1) vaccination against HPV and (2) screening for precancerous lesions. The introduction of the HPV vaccine has further contributed to a decrease in disease incidence. However, despite these preventive measures, cervical cancer persists as a significant global health concern [40,41]. 

Different cervical cancers carry different clinical implications and may require tailored treatment approaches based on their histological characteristics. In most cases, signs and symptoms of cervical cancer may not manifest during the precancerous stage of the disease. The primary method of detecting abnormal cells that could progress to cancer is through cervical screening tests. If precancerous changes progress to cervical cancer, signs and symptoms may include vaginal bleeding, pain during intercourse (dyspareunia), menstrual bleeding that is longer or heavier than usual (menorrhagia), bleeding after intercourse (postcoital bleeding), pelvic pain, changes in vaginal discharge, such as increased discharge or a discharge with a strong or unusual color or odor, and vaginal bleeding after menopause. Early detection and prompt treatment of cervical cancer can improve outcomes and increase the likelihood of successful treatment. Regular cervical screening tests are essential for early detection and prevention of cervical cancer [30,31,37,38,39,40,41].

## 4. Staging in Cervical Cancer

The clinical staging system universally adopted for cervical cancer is the International Federation of Gynecology and Obstetrics classification system. When accessible, PET/CT, CT, and MRI offer indispensable anatomical and metabolic insights, profoundly impacting a patient’s therapy and management [42]. 

### 4.1. Primary Tumor

Cervical carcinoma’s local extent is typically assessed through clinical examination under anesthesia. However, magnetic resonance imaging (MRI) has emerged as the preferred examination tool because of its superior resolution, enabling accurate diagnosis of tumor size, volume, and parametrial infiltration. MRI exhibits an accuracy range of 90–100%, surpassing the 60–70% accuracy rate of computed tomography (CT) [42,43].

Fluorine-18-labeled fluoro-2-deoxy-d-glucose positron emission tomography/computed tomography (FDG-PET/CT) plays a role in the initial diagnosis of primary cervical tumors, given their typically avid FDG uptake. FDG-PET’s ability to precisely detect primary tumors in the cervix has been demonstrated by different research studies. However, sensitivity may vary based on tumor stage and differentiation, with PET/CT showing higher accuracy compared to separate PET and CT scans [44]. Tumor volume also plays a crucial role as a predictive factor in cases of cervical cancer. Poor regression of initial tumor volume during treatment has been associated with lower overall survival rates [45]. FDG-PET/CT can provide solid information, particularly regarding involvement of lymph nodes and distant metastases. Tumor volume and FDG uptake serve as important prognostic factors, guiding treatment decisions and predicting outcomes in cervical cancer patients.

### 4.2. Nodal Staging

Nodal staging plays a primary role in determining disease outcome in cervical cancer patients. The involvement of lymph nodes significantly impacts prognosis, with a stark contrast in the overall survival rates between patients with a positive or negative status of the lymph nodes. For instance, individuals having small tumors and negative for lymph nodes have an overall survival rate of 90%, whereas those with positive pelvic lymph nodes exhibit almost 50% overall survival. Patients with positive para-aortic lymph nodes face an even bleaker prognosis, with an overall survival rate of less than 20–30% at 5 years [32,45].

Precise assessment of node status is essential for treatment, especially when radiotherapy is included. If metastatic lymph nodes are seen in the pelvic or para-aortic region, intensity-modulated radiation treatment with dose increase directed towards the affected area may be required. PET/CT has a 53–73% sensitivity and 90–97% specificity for identifying lymph node association in early-stage illness. The sensitivity and specificity of identifying para-aortic lymph node involvement rise to 75% and 95%, respectively, in more advanced stages (>IB2). For staging of lymph nodes, PET/CT has proven to be more accurate than CT. However, the illness stage affects FDG-PET/CT’s sensitivity and specificity [46]. There are reports that PET sensitivity is higher than MRI sensitivity. PET/CT sensitivities have been observed to vary [47].

In clinical practice, PET/CT findings have influenced treatment decisions, leading to modifications in treatment plans based on additional PET findings. Studies have demonstrated PET/CT’s ability to impact treatment outcomes compared to those with negative findings on CT scans alone [13]. Overall, PET/CT serves as a valuable tool for accurate lymph node staging in cervical cancer, aiding in treatment planning and prognostic assessment.

### 4.3. Distant Metastasis

Hematogenous tumors have the ability to spread to the liver, bone marrow, and lungs. PET/CT is reported to be more effective than MRI and CT for evaluating disease in individuals with advanced stage disease, lymph node metastases, and possible recurrent disease. Additionally, a correlation between osseous metastatic illness and lymph node metastases has been discovered. Loft et al. discovered that 10 out of 119 patients had metastatic disease, and they came to the conclusion that PET/CT has a sensitivity of 100%, specificity of 94%, positive predictive value of about 63%, and negative predictive value of 100% [7].

## 5. Screening and Diagnosis

Screening and diagnosis of cervical cancer involve many non-invasive imaging methods, typically used in active disease conditions, but they do not pinpoint the site of recurrence. Over the past few decades, improvements in medical imaging have raised the standard and effectiveness of medical diagnosis. Selecting various imaging modalities for distinct objectives aids in streamlining and improving the reach of one approach without necessarily replacing the others. PET scans, or positron emission tomography scans, are a common diagnostic tool. Biologically active tracers, which are often injected or consumed by the patient, are used in the setup. The biologically active tracer’s characteristics can be changed to meet the needs of the intended measurement. After that, a computer analyzes the measured data to create a contour plot of the relevant section of the body. These days, a different technique called computed tomography (CT) is also used [48,49].

## 6. Positron Emission Tomography (PET) and Computerized Tomography (CT)

According to the Society for Nuclear Medicine, molecular imaging refers to the identification, description, and measurement of biological processes occurring at the molecular and cellular levels in humans and other living things. Computed tomography (CT) and positron emission tomography (PET) have become necessary tools in clinical processes in oncology, with a wide range of uses from response assessment and surveillance to improved tumor staging and therapy planning. PET is widely used in many different fields of medicine. It is a diagnostic tool as well as a research tool. When used in clinical settings, PET can be used to detect neurological illnesses, brain tumors, and prostate and breast malignancies. PET/CT imaging is still based on radiolabeled glucose (^18^F-fluorodeoxyglucose (FDG)), although the technology is developing quickly. A wide range of radiolabeled probes are available, broadening the applications of PET/CT imaging. With the development of novel radiopharmaceuticals, the arsenal of PET imaging has been expanded to include targets for protein synthesis, lipid metabolism, hypoxia, and different biochemical pathways. In addition, freshly approved probes that are designed to target certain ligands, antigens, and receptors—like the prostate-specific membrane antigen (PSMA)—are making significant advances in precision medicine and are quickly being adopted in clinical settings [50]. The cutting-edge PET/CT hybrid imaging technique amalgamates data pertaining to the physiological or pathological distribution of a PET tracer with anatomical information, thereby enabling comprehensive visualization and analysis of molecular processes in vivo. 

These imaging techniques contribute significantly to identifying patients with recurrent cervical cancer. They enable precise visualization of tumor extent, metastases, and lymph node involvement, thereby aiding in treatment planning and prognostic assessment. However, it is important to note that while SCC levels serve as useful biomarkers for disease activity, imaging modalities are essential for localizing recurrence sites and guiding therapeutic interventions. CT remains a common imaging modality in patients diagnosed with cervical carcinoma, especially for identifying lymph node enlargement or extensive disease beyond the cervix [51,52,53]. However, CT has demonstrated limitations in accurately assessing parametrial involvement or tumor size due to inadequate contrast between the local tumor and the parametrium [54,55,56,57]. Initial findings suggest that MRI surpasses CT in delineating cervical tumor boundaries and measuring tumor size with great accuracy [8,9,10,17,58,59,60,61,62,63,64,65,66,67,68].

Positron emission tomography stands as a prominent functional imaging modality extensively employed in contemporary clinical diagnostics across a diverse spectrum of ailments. Utilizing positron-emitting isotopes with brief half-lives, such as carbon-11 (half-life of 20.4 min) and fluorine-18 (half-life of 109.7 min), PET facilitates the in vivo assessment of physiological processes. Beyond its clinical utility, PET serves as a pivotal tool in preclinical investigations involving animal models and in identifying specific biomolecules within the human body. 

The combined PET/CT scan represents a pivotal advancement allowing for simultaneous imaging of both anatomical and metabolic information. This integrated approach is swiftly emerging as an indispensable diagnostic tool for delineating therapeutic strategies for malignant lesions [11,12]. In CT/PET research studies reported so far, generally favorable patient-based sensitivity rates range from 73–77%, specificities range from 56–97%, and accuracies range from 68–89% in cervical cancer [69]. Similarly, in endometrial cancer, sensitivities have been documented at 50–63%, specificity at 87%, and accuracy at 78–83%. In a comparison of FDG-PET alone, contrast-enhanced CT alone, and FDG-PET/contrast-enhanced CT, PET/CT performed better than FDG-PET alone, showing a 10% improvement in sensitivity, 20% improvement in specificity, and accuracy of 16% [70].

However, caution must be exercised during certain periods such as menstruation and postmenopausal hormonal therapy as these can influence PET scan results. Conditions such as hepatocellular cancer, scirrhous stomach cancer, bronchovesicular carcinoma, and urinary tract malignancies might result in false-negative results. In addition, tumor size of less than 1 cm may yield false-negative results due to the limited spatial resolution (4–5 mm) [71].

The standardized uptake value (SUV) in PET serves as a semi-quantitative index, representing the ratio of the tracer within a region of interest to the tracer’s overall concentration, under the assumption of uniform tracer distribution and non-elimination. However, corrections based solely on body weight may lead to overcorrection, particularly in individuals with high body fat. Furthermore, SUVs are influenced by the setting of the ROI, reflecting the non-uniform distribution of tissues within a tumor and varying glucose metabolism. The selection of either the maximum-pixel SUV or the mean SUV within the ROI can significantly impact SUV calculations. Nonetheless, an SUV ranging from 2.5 to 4.0 or higher is generally indicative of a malignant lesion. SUV has gained increasing significance as numerous studies have identified it as a prognostic factor in cancer within the context of FDG-PET/CT imaging. SUVmax, which indicates the level of FDG activity in a primary tumor, is a prognostic biomarker for the condition of the lymph nodes and the course of the disease. Cervical cancer differentiation and tumor histology influence FDG uptake, with well-differentiated and squamous tumors exhibiting lower SUVmax values [45]. CT/PET is a vital modality in the staging of cervical cancer [72].

## 7. PET-Based Radiopharmaceuticals

The utilization of radiopharmaceuticals as medicinal agents for in vivo diagnostic and therapeutic purposes dates back to the 1960s. These substances consist of two structures: a radionuclide that emits positrons and a molecular framework that is known as a carrier molecule. When the radioisotope is injected into the body as a radioactive tracer, it attaches itself to the carrier molecule, also known as a ligand. Given the short half-lives of these emitting isotopes, their production necessitates facilities equipped with in-house cyclotron capabilities. PET/CT represents a cutting-edge nuclear imaging methodology particularly valuable for probing the functional aspects of cancer pathology. This sophisticated technique enables the comprehensive examination of various organs including the thyroid, bones, heart, liver, and others, facilitating the detection of abnormalities in their physiological processes through the utilization of PET radiopharmaceuticals. Notably, the majority of positron-emitting radioisotopes possess fleeting half-lives, necessitating the establishment of in-house cyclotron facilities for their production [73,74].

The progression of radiopharmaceutical compounds targeting specific diagnostic and therapeutic goals, pivotal for both imaging modalities, advances concurrently with the development of acquisition systems. When selecting a PET radionuclide, several critical factors must be taken into account, foremost among them being radionuclide availability, followed by its physical characteristics, as well as radiochemical and radiopharmacological considerations. 

A diverse range of PET radiopharmaceuticals have undergone rigorous evaluation in clinical trials, spanning a broad spectrum of diseases. All these PET compounds have strict requirements to meet as imaging agents, even though their vehicle molecules (or ligands) differ. High specificity, strong binding affinity, low toxicity, stability (especially against different enzymes in plasma), quick clearance from non-targeted tissues, affordability, and approval for clinical use are some of these requirements.

The selection or development of a radiopharmaceutical necessitates meeting specific criteria to ensure precise biological targeting or disease diagnosis. Notably, the radionuclide employed must possess an appropriate half-life commensurate with the intended application. Moreover, factors such as molecular size or charge, stability, lipophilicity, metabolism, and specific activity of the labeled compounds significantly influence the targeting specificity of each biological target. Therefore, comprehensive quality control encompassing radiochemical, physicochemical, and biological parameters is imperative [75]. There are a number of radiopharmaceuticals employed based on PET for imaging of cervical cancers and related hypoxia as presented in Table 2.

A notable correlation has been observed between low oxygen partial pressure and elevated uptake of [^64^Cu]-ATSM. Nonetheless, preclinical evidence has shown that [^64^Cu]-ATSM may not universally serve as a hypoxia marker across various tumor types. The interaction between [^64^Cu]-ATSM and the upregulation of fatty acid synthase has been specifically linked to prostate cancer, notwithstanding relatively low levels of [^64^Cu]-ATSM uptake induced by heightened hypoxic environments. In patients with cervical cancer, [^64^Cu]-ATSM has shown potential as a marker [77].

## 8. FDG-PET and Cervical Cancer Diagnosis

The PET radiopharmaceutical considered as the gold standard is [^18^F]-fluorodeoxyglucose ([^18^F]-FDG), a compound that can be taken up by cancerous cells, and advancement of radiotherapy techniques has enabled the delivery of high dose rates (HDRs) to tumors located in close proximity to normal tissues, while simultaneously sparing these healthy structures through precise dose sculpting. Traditionally, anatomical images have been utilized for treatment planning; however, they often lack the sensitivity required to accurately define tumor extent and evaluate the biological characteristics of both the tumor and normal surrounding tissues [75]. In this perspective, the integration of biological imaging with anatomical images is crucial. Biological imaging techniques enable the mapping of molecular dispersals within the tumor and adjacent tissues. By incorporating these biological images into treatment planning, clinicians can better understand the biological behavior of the tumor and surrounding tissues, allowing for more precise and personalized external beam radiotherapy. This integration facilitates improved targeting of tumor cells while minimizing radiation exposure to healthy tissues, ultimately enhancing treatment efficacy and reducing potential side effects. Diagnostics utilizing PET imaging can be performed using radiotracers labeled with ^15^O, ^18^F, and ^11^C. Among all these radiotracers, FDG exhibits notable accumulation in normal tissues characterized by heightened glucose metabolism and is internalized into cells via a glucose transporter. When FDG enters cells, it is phosphorylated to produce FDG-6-phosphate by hexokinase. This compound has low membrane permeability and slow metabolism in glycolysis, which prevents it from diffusing out of the cells. As a result, FDG builds up in areas that consume a lot of glucose, like brain tissue, heart muscle, and tumors, where glucose-6-phosphatase activity is low. However, FDG is fundamentally non-specific, with non-malignant processes demonstrating FDG absorption, including inflammation, colonic and gynecologic activity, thymic hyperplasia, brown fat, and organs with high glucose utilization, such as the brain and liver. FDG is primarily eliminated through the renal or urine system, in contrast to glucose [84]. The literature lists about 4000 chemicals conjugated with positron-emitting radionuclides, but only a few are routinely used in clinical practice [85,86].

PET imaging is appealing because tumor cells actively ingest radiolabeled fludeoxyglucose. Early investigations on PET in cervical cancer patients indicated that it outperformed CT in detecting illness in pelvic and abdominal lymph nodes. However, these studies were limited by their small sample sizes, retrospective nature, and lack of evaluation regarding the clinical impact of PET. Subsequent advancements included the combination of CT with PET to integrate anatomical structure with functional imaging. Despite this progression, limited evidence existed regarding the clinical benefits or improved outcomes associated with PET/CT. Nevertheless, PET/CT gradually gained acceptance in various jurisdictions [87,88].

An important pathological characteristic of cervical cancer is associated tumor hypoxia, denoting insufficient oxygen levels within cells, serving as a prognostic indicator. Hypoxia’s relevance extends to cancer management, influencing responses to radiation therapy chemotherapy [50,89,90,91]. Furthermore, hypoxia plays a pivotal role in predicting metastases due to its association with DNA mutations and the proliferation of malignant, atypical cells [92].

## 9. Diagnostic Strategies at Various Stages of Cervical Cancer

### 9.1. Primary Tumor Diagnosis

FDG-PET has low efficacy in screening and is considered as not appropriate for imaging early-stage cervical cancer. Diagnostic imaging plays a modest role in cervical cancer care centers because of the uncomplicated nature of examination and cytodiagnosis in the uterine cervix, where early detection is critical for therapeutic outcomes [93]. PET demonstrated high sensitivity ranging from 85–92% for detecting primary lesions in patients with stage Ib, with the ability to identify the majority of primary tumors measuring 2 cm or bigger. In research by Ohno et al., preoperative FDG-PET cervical cancer patients had primary tumors of intraepithelial, minimally invasive, and invasive cancer types, although aberrant accumulation could not be detected in cases categorized as stage Ib or IIa [94,95]. PET can also identify endocervical adenocarcinoma. Nonetheless, these findings collectively indicate that PET is ineffective in detecting tiny lesions, particularly early-stage cancer due to limited spatial resolution [96].

### 9.2. Metastases of Lymph Node Diagnosis

Lymph node metastasis is critical for early detection and treatment, and it serves as a key prognostic factor in cervical cancer. The most reliable diagnostic and therapeutic modality is systematic lymph node dissection; nevertheless, due to its technical difficulties and related postoperative risks, non-invasive detection methods for lymph node metastases are required. Conventional imaging techniques such as CT and MRI may detect lymph node enlargement indicative of metastasis, yet their diagnostic accuracy remains unsatisfactory [15]. In contrast, multiple studies have demonstrated the efficacy of PET in detecting lymph node metastases. A meta-analysis found that FDG-PET has a high sensitivity and specificity for preoperative identification of pelvic lymph node metastases, outperforming CT and MRI.

In cervical cancer scenarios, FDG-PET/CT shows promise for identifying lymph node metastases. Studies have demonstrated improved sensitivity when compared to MRI, as well as robust performance in detecting both pelvic and para-aortic lymph node metastases. The addition of FDG-PET/CT findings in the staging parameters is expected to improve preoperative diagnostic and treatment planning accuracy. Despite its advantages, FDG-PET/CT can produce false-negative results, emphasizing the significance of meticulous lymph node dissection in treatment planning, especially in advanced cancer cases. Prospective investigations have shown that lymph node metastases discovered with FDG-PET/CT can serve as predictive indicators for recurrence-free survival, underscoring the clinical utility of FDG-PET lymph node staging. Nevertheless, further prospective investigations are warranted to address concerns regarding false-negative findings associated with FDG-PET/CT [7,13,16,97].

### 9.3. Recurrent Lesion Detection

Diagnosing pelvic recurrence with CT and MRI presents difficulties, especially after surgery, because these modalities may fail to accurately identify the anatomical position or detect fibrosing, necrotic lesions, or inflammatory alterations. However, combining data of CT/MRI with PET showed evidence of FDG uptake that results in improvement of detection of recurring lesions.

A study on FDG-PET/CT for evaluating recurrent cervical cancer indicated that the accuracy, specificity, sensitivity, and negative and positive predictive value were all around 96% for detecting local recurrence at the initial site. The study found that distant metastases could be detected at 96%, 95%, 95%, 96%, and 95% accuracy. These findings emphasize the remarkable efficacy of PET/CT in detecting recurrent cervical cancer and localizing distant metastases, suggesting its great clinical relevance in this context [98].

## 10. Present Status of ^18^F-FDG PET

Presently, one PET tracer, ^18^F-FDG, holds approval for routine clinical use. Its applications span across oncology, cardiology, and neurology, with oncological utilization being predominant. ^18^F-FDG PET capitalizes on the premise that diseased or tumor cells exhibit heightened glucose metabolism, yielding higher signal intensities compared to normal tissues and background levels. A notable advantage of ^18^F-FDG PET is found in its ability to detect metastatic disease. However, inflammation and infection can also cause uptake up ^18^F-FDG, posing significant confounding factors in PET imaging. Moreover, the efficacy of ^18^F-FDG in monitoring early responses to therapy remains constrained. Technological advancements have fostered the realization of personalized medicine, with tracer targets and disease control encompassing multifaceted dimensions. The ideal tracer would serve as a precise probe tailored for specific targets, processes, and temporal contexts, thus optimizing patient care. A noteworthy approach to disease control involves monitoring of disease progression and judicious utilization of cancer therapeutics. Common targets for such interventions include metabolism, inflammation and infection, proliferation, hypoxia, angiogenesis, and apoptosis. The first two targets, in particular, have been extensively explored through ^18^F-FDG PET and are firmly integrated into routine oncological practices. Contrary to the concept of cognitive fusion, an alternative method involves utilizing a single-scan simulator, where the radiation therapy arrangements for CT and PET scans are acquired simultaneously, with the patient positioned on a flat couch for treatment. The fusion process occurs automatically, leveraging a shared coordinate system between the CT and PET images. PET employs uptake of ^18^F-labeled fluorodeoxyglucose to identify metabolically active tissues indicative of malignancy. PET/CT integrates PET and computed tomography to provide anatomical imaging. CT images aid in localizing and characterizing abnormal metabolic activity detected as a result of PET scans, thereby enhancing the scan for analysis and interpretation. However, CT scans performed in conjunction with integrated PET/CT examinations often follow a low-dose strategy, with lower exposure factors and homogeneous scanning parameters [14].

## 11. Therapeutic Strategies to Manage Cervical Cancer

Treatment for early-stage cervical cancer (stages I–IIA) typically involves surgical intervention or chemoradiation, while advanced-stage cervical cancer (IIB–IIIB) is primarily managed with chemoradiation. Chemotherapy as a standalone treatment is reserved for cases of metastatic cancer at the time of presentation. Recurrence rates are higher in advanced cervical cancer with the presentation rate of 30% compared to early-stage cervical cancer, with a rate of 6% [55,99]. Presently, surveillance for recurrence relies on regular clinical examinations during follow-up visits. In instances where recurrence is suspected based on symptoms or clinical findings, confirmation and assessment of the recurrence’s extent are typically achieved through CT or MRI. Notably, neither imaging modality can reliably differentiate between radiation-induced fibrosis and malignant recurrence.

Depending on the treatment type, a team may consist of a number of different health professionals, especially radiation and medical oncologists, who decide and prescribe the course of chemotherapy. The therapeutic approach is contingent upon the staging of the ailment. In instances characterized by early-stage and non-bulky pathology (measuring less than 4 cm), surgical intervention constitutes the primary treatment modality, oftentimes complemented by adjunctive chemoradiation therapy. In scenarios where the lesion is small, a cone biopsy may present as a viable therapeutic option; however, certain circumstances necessitate the execution of hysterectomy, entailing the surgical extirpation of the uterus. Conversely, for cases exhibiting locally advanced pathology, a therapeutic regimen comprising the synergistic application of radiation therapy, encompassing radiotherapy modalities, and chemotherapeutic agents, such as cisplatin, is implemented. In the context of metastatic disease, the therapeutic paradigm is primarily anchored upon chemotherapy, incorporating platinum-based agents, in conjunction with fluorouracil, or may pivot towards the provision of palliative care as a standalone measure.

Exenterative surgery, which involves the removal of the bladder, uterus, vagina, and/or rectosigmoid in a properly selected sample with pelvis-confined or central recurrence, has the potential to cure. The long-term impact on the woman, including psychosocial consequences, are found to be significant. Accurately triaging women with distant metastases to palliative care and women with possibly curable central pelvic recurrence to exenterative surgery is crucial in the treatment of recurrent cervical cancer. It is fair to expect that improved early diagnosis of recurrence in asymptomatic women will increase survival by identifying women with pelvis-confined recurrence with a high risk of mortality and morbidity who can undergo salvage surgery. The incidence of cervical cancer has shown a significant decrease since the introduction of the National Cervical Screening Program in 1991 and the introduction of a national human papillomavirus (HPV) vaccine program in 2007. These preventive measures have played a pivotal role in reducing the frequency of cervical cancer by targeting both early detection through screening and primary prevention through vaccination against HPV, the primary cause of cervical cancer [100].

## 12. Follow-Up of Treatment Response and Disease Recurrence

Follow-up PET scans can serve as invaluable tools for physicians overseeing clinical trials of novel therapies. These scans hold predictive value for long-term survival outcomes. By utilizing PET scans, clinical researchers can obtain early insights into the potential effectiveness of experimental treatments. 

Preoperative prognostic prediction using FDG-PET/CT holds significant promise in tailoring therapeutic strategies. Kidd et al. conducted a study of 287 patients, assessing pretreatment SUVs, and found SUV to be the only significant independent predictor related to overall survival. Notably, they found a 5-year overall survival rate of 95% [101].

In locally advanced cervical cancer, patients are at a high risk of disease recurrence, with roughly one-third reporting recurrence within two years of completing therapy. Clinical stage, lymph node status upon diagnosis, and tumor response following treatment are all risk factors for recurrence. The presence of fluorodeoxyglucose (FDG) activity, whether ongoing or new, can predict survival outcomes. Schwarz et al. found that metabolic response predicted long-term survival in research that used FDG-PET/CT three months after therapy completion. Patients with a complete metabolic reaction had a 3-year survival rate of 78%, compared to 33% for those with a partial metabolic response. Patients with advancing illness had a 0% survival rate. MRI scans can be used to assess tumor regression following external beam radiation therapy and chemotherapy. Regression was found to be less than 20% of residual tumor volume which significantly lowered the probability of local recurrence when compared with higher residual volumes [35].

### 12.1. Tumor Hypoxia

Among the important pathological aspects of cervical cancer, tumor hypoxia is particularly prominent [48]. Hypoxia is a lack of oxygen within cellular contexts that has substantial prognostic value. Its relevance extends to therapeutic techniques in cancer treatment, including reactions to chemotherapy or radiotherapy [50,102]. Hypoxia’s relationship with the prediction of metastatic dissemination in tumor cells is a cause for concern. Given these findings, assessing hypoxia is crucial in treatment regimens, especially for locally advanced stages and local recurrences, which are disproportionately common in cervical cancer cases. Radiolabeled hypoxia imaging agents are divided into two classes: diacetyl-bis(N4-methylthiosemicarbazone) (ATSM) analogues and nitroimidazole derivatives [92]. Among the nitroimidazoles, [^18^F]FMISO has seen broad use in recent clinical studies, enabling the detection of hypoxic tumor subvolumes and following their spatiotemporal changes [103,104]. It has been shown that [^18^F]FDG PET/CT is the preferred technique for both initial staging and ongoing cervical cancer patients’ surveillance.

In sixteen patients with histologically proven locally advanced cervical cancer, [^18^F]FDG/[^18^F]FMISO PET/MRI scans demonstrated the viability of this modality in cervical cancer cases [105]. These scans gave additional insights into tumor biology and heterogeneity, as well as the identification of hypoxic tumor subvolumes that were resistant to treatment modalities. [^18^F]FDG, [^18^F]FMISO, and [^18^F]FAZA have been evaluated in clinical trials, showing extensive functional correlations [106]. Studies have introduced a new hypoxia imaging agent named [^18^F]F-flortanidazole ([^18^F]HX4) which revealed its potential advantages over [^18^F]FMISO and [^18^F]FAZA, including faster clearance rates, higher image contrast, and lower background signals. Nonetheless, [^18^F] HX4 also exhibited higher variability among patients in terms of clearance and contrast. Alternative hypoxia imaging agents, such as ([^18^F]FAZA) and [^18^F]F- ([^18^F]FETNIM), have different pharmacokinetics than [^18^F]FMISO. Notably, [^18^F]FETNIM has depicted reduced tumor-to-non-target accumulation in lung cancer patients. Meanwhile, [^18^F]FMISO has a high lipophilicity, which allows for passive diffusion across cell membranes, which also leads to sluggish clearance and an unsatisfactory tumor-to-normal tissue ratio. In contrast, lower lipophilicity has been found [78,79,106,107,108,109].

### 12.2. Tumor Angiogenesis

Targeting the angiogenic system has emerged as an increasingly important treatment option for cervical cancer [100]. The choice of agents and combinations is important for understanding of cancer biology, the accessibility of anticancer medicines, and their associated toxicities. Integrin αvβ3 is overexpressed in tumor and angiogenic endothelial cells, making it an interesting therapeutic target. Cervix cancer patients showed β3 integrin expression has a considerable impact on prognosis, as evidenced by both univariate and multivariate analysis [110]. Studies indicate that αvβ6 expression in cervical cancer correlates with lower overall and disease-free survival rates. Increased expression of αvβ6 in cervical squamous carcinomas may indicate a poor prognosis. This could be due to activation of TGF-β1 at the tumor/stroma interface and an increase in the migratory capacity of αvβ6-expressing tumor cells [111].

Tumor-associated vasculature produces integrin αvβ3, which may help angiogenic cells attach to provisional matrix proteins in the tumor microenvironment and these sticky contacts may influence a variety of cancer-related processes [112,113,114]. As a result, there is a growing emphasis on developing better vascular imaging techniques to screen treatment efficacy, with significant efforts focused on characterizing integrin antagonists for their great capacity to selectively deliver diagnostic agents to tumor cells and associated blood vessels. In such circumstances, the use of FDG-PET/CT in selected individuals may allow for salvage curative therapy for local or oligometastatic disease. Notably, FDG-PET/CT was more effective in detecting true-positive results in symptomatic patients than in asymptomatic patients, indicating its potential relevance in directing salvage treatment.

## 13. Discussion

Nuclear medicine refers to the diagnosis and treatment of diseases using radiolabeled chemicals known as radiopharmaceuticals. Radiopharmaceuticals used in PET/CT and SPECT/CT have proven to be very reliable in diagnostic imaging in cancer imaging and therapy. The combination of nuclear medicine and radiological modalities provides additional information critical for diagnosis, staging, treatment regimen management, and assessment of therapeutic response. As a result, nuclear medicine plays a critical role in the clinical evaluation of oncological cancers. Furthermore, FDG is secreted by the kidneys and accumulates in the bladder, complicating the diagnosis of bladder tumors [14,115]. Despite FDG’s extensive clinical use, not all tumors show a significant increase in metabolism detectable with FDG-PET imaging. Prostate cancer, neuroendocrine tumors, and hepatic tumors, in particular, may have very low FDG uptake, making them almost invisible on PET scans. Furthermore, evaluating malignant lesions in tissues with physiological FDG uptake or excretion presents difficulties, as does discriminating between inflammation and malignancy. As a result, various other tracers have been proposed as a complement to FDG imaging [116,117]. The emergence of targeted medicines has posed hurdles to established ways of measuring tumor response to treatment, as many novel drugs are believed to generate cytostasis rather than cytotoxicity. As a result, tailored PET chemicals have been produced for oncological studies. Despite significant differences in tumor size estimation and genuine tumor response, PET has emerged as the most sensitive imaging method for assessing the metabolic activity of individual tumors [118].

PET provides various advantages, including the ability to perform whole-body scans in a single session while maintaining safety and with little invasiveness. Nonetheless, disadvantages include a lack of precise anatomical localization information and very low spatial resolution (about 3–5 mm), which limits the detection of tumors smaller than about 10 mm. Initially thought to be unsuitable for gynecological tumors due to proximity to the bladder and artefacts caused by the ovaries, advances in image processing technology and the introduction of PET/CT have mitigated these issues, considerably improving diagnostic accuracy in this field. The use of FDG PET/CT is important in properly delineating radiotherapy volumes, protecting active bone marrow from excessive radiation doses, and enabling precise brachytherapy delivery. Despite better survival rates from chemoradiotherapy (CRT), establishing optimal loco-regional control remains a substantial issue, necessitating additional treatment modalities. Advances in understanding the tumor microenvironment, which includes variables such as hypoxia and angiogenesis, give prospects for applying innovative molecular targeted therapies. Advances in biological imaging modalities, such as PET/CT, have a significant impact on evaluating treatment responses to novel therapy regimens.

## 14. Conclusions

In conclusion, PET/CT has evolved and is widely used for disease detection, prognosis, and therapy planning. FDG-PET/CT is the most reliable method for detecting metastatic lymph nodes in cervical cancer, playing a significant role in staging and treatment planning. However, its limitations highlight the need for additional research and refining in this area. PET/CT can improve radiation treatment planning by allowing for more targeted therapy with little impact on nearby structures. Combining PET measurements with PET/MRI parameters improves illness prognosis and therapy planning. FDG-PET/CT provides the early discovery of resectable recurrence, allowing physicians to modify the planned therapy. According to FDG-PET/CT data, surgical excision of limited recurrent disease increased patients’ survival rates with recurrent cervical cancer. Further research is required for cervical cancer management, especially on diagnostics and therapeutics, depending on the stage of disease.

## 15. Future Directions

PET imaging provides very valuable information about disease occurrence, therapeutic response, and recurrence management. For cancer or tumor imaging, future applications of PET/CT will rely on strong reproducible quantitative data. The published research and literature have proved the benefits of PET/CT applications but organization and evaluation of the significance of biomarkers are still required in the field of oncology. Research is required on the development of new radiotracers as diagnostic agents for tumors with minimal FDG uptake in different cancers. Several cancer therapies employ specific agents targeting receptors or enzymes in tumor cells. This strategy can be employed for the development of new PET/CT imaging agents. The proper route to validate the abilities of new radiotracers for cervical cancer and their approval through the FDA and other medical organizations must be processed and supported.

## Figures and Tables

**Table 1:** Various imaging modalities related to cervical cancer.

Imaging Modality	Procedure	Advantages	Drawbacks	References
CT	CT uses X-rays from different angles after taking a shot of dye before the X-rays and computer-based images	Short time required for scan Accessible at almost all radiotherapy centers Variety of materials can be employed Clear visibility of image	Uterine and cervical borders may be delineated	[6,7,8,9,10,11,12]
CT/PET	Injection of a small amount of [^18^F]FDG. Cancer cells take it and PET scan shows location of radioactive tracer in body	Cervical cancer staging Detection of recurrent cervical cancer Advantage related to biochemical changes associated with disease/cancer	False negatives with lesions less than 0.5 cm False-positive results can be observed with chronic inflammation	[13,14,15,16]
MRI	In MRI scans, contrast material is injected through intravenous administration. Scan lasts from 15 min to more than an hour	Perfect for resolution of soft tissues Good for delineation of tumor tissue Can differentiate the tumor boundaries	High cost and long time Presence of metals in the patient’s body makes MRI unsuitable MRI machines are not available everywhere	[8,9,15]
Ultrasound	Transvaginal ultrasound takes into consideration high-energy sound waves that bounce off various tissues and organs, leading to diagnosis	Can diagnose pelvic masses or cervical problems Low cost and availability everywhere Tumor visibility clear	Unable to diagnose tumor volume precisely Lack of treatment prediction Poor clinical evidence	[2,5,17]

**Table 2:** PET-based radiopharmaceuticals and their uses in diagnostics and therapy.

PET Radiopharmaceutical	General Remarks	Purpose	References
[^18^F]FDG	Gold standard of PET used in oncology, neurology, and cardiology	Imaging	[76]
[^64^Cu]-ATSM	Predictive predictor of tumor response to treatment	Indicator of tumor response to therapeutics in cervical cancer	[77]
[^18^F]FDG	This modality is feasible in hypoxic lesions of cervical cancer	Cervical cancer	[78]
[^18^F] FMISO	Gold standard for hypoxic tumor volume detection	Hypoxia imaging	[79]
[11-C]Acetate	Originally employed in cardiology, it is now often used in prostate cancer imaging	Imaging	[80]
[^18^F]-HX4	The reliability of [^18^F]-HX4 PET has been assessed. A strong relationship between first and second HX4 PET	Cervical cancer imaging	[81]
[^18^F]HX4-cervix	Assessing tumor hypoxia to investigate optimal [^18^F]-HX4 uptake time	Cervix imaging	[81]
^18^F-FDG	2-floro-2-deoxyglucose-based assessment of increasedglucose metabolism by overexpression of GLUT-1	Oncology	[42]
[^18^F]FES	Observation of binding ability to ER	Imaging by estrogen receptor expression in gynecologic cancers	[82]
[^18^F]4FMFES	Estrogen receptor (ER) binding	Imaging of gynecologic malignancies	[82]
^68^Ga-FAPI-04	Comparison the SUVmax of ^18^F-FDG and ^68^Ga-FAPI-04 PET/CT for primary lesions in cervical cancer was made	Cervical cancer	[83]

## Abbreviation

PET	Positron emission tomography
CT	Computed tomography
FDG	^18^F-fluoro-d-glucose
SUV	Standardized uptake value
MRI	Magnetic resonance imaging
HPV	Human papillomavirus
PSMA	Prostate-specific membrane antigen
SCC	Squamous cell carcinoma
SUVmax	Maximum standardized uptake value
HDRs	High dose rates
[^64^Cu]-ATSM	^64^Cu-diacetyl-bis (N^4^-methylthiosemicarbrazone)
[^18^F]FES	16α-[^18^F]-fluoro-17β-estradiol
CRT	Chemoradiotherapy
[^18^F]4FMFES	4-fluoro-11β-methoxy-16α-^18^F-fluoroestradiol
^68^Ga-FAPI-04	^68^Ga-fibroblast activation protein-specific inhibitor
[^18^F]FMISO	^18^F-fluoromisonidazole
[^18^F]FAZA	^18^F-fluoroazomycin arabinoside
([^18^F]HX4)	[^18^F]F-flortanidazole
[^18^F]FETNIM	[^18^F]Fluoroerythronitroimidazole
CRT	Chemoradiotherapy
